# First clinical utility of sensing Ultrasound Localization Microscopy (sULM): identifying renal pseudotumors

**DOI:** 10.7150/thno.100897

**Published:** 2025-01-01

**Authors:** Sylvain Bodard, Louise Denis, Georges Chabouh, Dany Anglicheau, Olivier Hélénon, Jean-Michel Correas, Olivier Couture

**Affiliations:** 1 AP-HP, Hôpital Necker Enfants Malades, Service d'Imagerie Adulte, Université de Paris Cité, F-75015, Paris, France.; 2 Sorbonne Université, CNRS, INSERM, Laboratoire d'Imagerie Biomédicale, Paris, France.; 3 Department of Radiology, Memorial Sloan Kettering Cancer Center, 1275 York Avenue, New York, NY 10065, USA.; 4 Center for Transplantation Sciences, Massachusetts General Hospital, Harvard Medical School, Boston, Massachusetts, USA.; 5 AP-HP, Hôpital Necker Enfants Malades, Service de néphrologie-transplantation rénale adulte, Université de Paris Cité, F-75015, Paris, France.

**Keywords:** ultrasonography, sensing ultrasound localization microscopy, kidney neoplasms, kidney glomerulus, pseudotumor

## Abstract

**Rationale:** Renal pseudotumors, which mimic tumors on imaging, pose diagnostic challenges that can lead to unnecessary interventions. Sensing ultrasound localization microscopy (sULM) is an advanced imaging technique that uses ultrasound imaging and microbubbles as sensors to visualize kidney functional units. This study aims to investigate whether sULM could differentiate between renal pseudotumors and tumors based on the presence of glomeruli.

**Methods:** Eleven patients (6 tumors, 6 pseudotumors - 1 patient with 2 pseudotumors) were included. Data on patient demographics, tumor characteristics, and sULM metrics were collected. Glomeruli were quantified and compared among tumors, pseudotumors, and renal cortex using sULM. Additional metrics, i.e., normalized speed and dispersity, were also analyzed.

**Results:** Renal tumors exhibited fewer detected glomeruli paths (mean: 10 ± 6 /cm^2^ [range: 4-20]) compared to pseudotumors (26 ± 5 /cm^2^ [19-32], p < 0.001) and normal renal cortex (26 ± 6 /cm^2^ [15-35], p < 0.01). Tumors displayed lower dispersity (0.13 ± 0.06 arbitrary units [a.u.] [0.07-0.20]) than both the renal cortex (0.3 ± 0.1 a.u. [0.1-0.4], p = 0.0012) and pseudotumors (0.22 ± 0.05 a.u. [0.16-0.25], p = 0.0389), and lower normalized speeds of 0.08 ± 0.04 without units (w.u.) [range: 0.03-0.17] compared to the renal cortex (0.18 ± 0.07 w.u. [0.11-0.28], p = 0.0014) and pseudotumors (0.14 ± 0.02 w.u. [0.12-0.16], p = 0.0497). sULM could effectively differentiate renal pseudotumors from tumors based on glomerular detection and metrics estimation.

**Conclusion:** This initial exploration into the clinical utility of sULM suggests it could provide a noninvasive tool to support patient management, particularly for individuals with contraindications to conventional imaging methods. Further studies are needed to confirm these preliminary findings.

## Introduction

Renal pseudotumors, composed of nonneoplastic tissue, can display similar behavior to tumors on clinical imaging 1-3. Though usually diagnosed by contrast-enhanced computed tomography (CT) or magnetic resonance imaging (MRI) 1, they can pose a challenging diagnosis and may sometimes require biopsy. They can be categorized as developmental, infectious, granulomatous, or vascular based on their varied and distinct origins 1. The most frequent developmental renal pseudotumor is the hypertrophied column of Bertin 4 which manifests as cortical renal tissue pushing into the pelvis between the medullary pyramids 5. Additionally, focal compensatory hypertrophy, another variant of renal pseudotumor, can closely resemble the hypertrophied column of Bertin 6.

Ultrasound localization microscopy (ULM) 7-9 is an acoustic super-resolution technique that tracks intravascular ultrasound contrast agents (microbubbles) to map an organ's microcirculation 10,11. This technique has achieved unprecedented resolution in living animals 12-15 and human organs 16-19. While ULM provides detailed microvascular maps, it cannot visualize the functional units within organs. However, sensing ULM (sULM) is a substantial technological enhancement in ULM technique that utilizes microbubbles as sensors of their immediate environment to visualize organ functional units. By classifying microbubble tracks based on an expected behavior predicted from microanatomy knowledge, sULM has successfully observed the kidney's glomeruli in renal graft 20 and native kidneys 21 by highlighting microbubbles swirling at low speed within a confined capillary bundle. This represents the first time that an imaging technique has been able to visualize glomeruli *in vivo*, as no other conventional imaging techniques can achieve this due to the small size of the glomeruli 20.

Given that tumors are not populated by glomeruli, sULM has the potential to differentiate pseudotumors from tumors by observing the presence of glomeruli. This clinical application could be useful for patients who cannot benefit from enhanced CT scan or MRI, such as patients presenting with severe chronic renal failure or who are at risk of biopsy complications, respectively 22-24. This study's objective was to investigate whether sULM could differentiate pseudotumor from tumor based on the presence or absence of glomeruli. A secondary objective was to test whether microcirculation, explored via dispersity and normalized speed, could provide additional arguments.

## Methods

### Ethics approval

This study was approved by the Ethics Committee of the French Society of Radiology (CERF, reference number CRM-2304-336). Patient recruitment occurred in our genitourinary university center.

### Population study

From January 1 to March 30, 2023, 14 patients with 8 proven renal tumors and 7 renal pseudotumors in whom a definitive diagnosis could not be established using standard ultrasound and clinical contrast-enhanced ultrasound (CEUS), were retrospectively included. Three patients were excluded due to the unavailability of the CEUS cine loop (2 patients had 1 tumor, and 1 patient had 1 pseudotumor). A total of 6 renal pseudotumors (5 patients, including 1 with 2 pseudotumors) and 6 renal tumors (6 patients) composed the study population.

**Table S1** summarizes the standard ultrasound and CEUS manifestations of the lesions.

**Figure S1** summarizes the flowchart of the study.

### Data collection

Data collection encompassed demographic, tumoral, and CEUS parameters. Demographic information, including age, sex, body mass index (BMI), and estimated glomerular filtration rate (eGFR) was recorded for each participant. Lesion (tumor and pseudotumor) characteristics collected included tumor size, kidney side, maximum diameter (as determined by ultrasound), diagnostic methods, and radiographic evaluation of the renal masses score 25. CEUS data included tumor depth, frame rate, and loop duration.

All data were anonymized and subsequently analyzed with MATLAB (version R2002a, MathWorks Inc.; Natick, MA, USA).

### Gold standard

The gold standard for tumors was pathological analysis (on biopsy or surgical specimen) or typical CT features for angiomyolipoma. For pseudotumors, the gold standard was pathological analysis (on biopsy) or concordance from CT scans and MRI examination when unavailable. **Figure S2** shows an example of diagnosis (gold standard) provided by imaging.

### CEUS acquisition

CEUS were performed using a clinical ultrasound scanner Aplio i800 (Canon MS; Nasu, Japan) and a convex abdominal probe i8cX1 (3MHz, Canon, bandwidth [1.8-6.2] MHz), with focused sectorial beams which decrease significantly the frame rate, i.e. from 39 to 44 images per second. Patients were positioned in the lateral decubitus position and held their breath during the acquisition. A bolus of 1.2mL of contrast agent (SonoVue®, Bracco; Milan, Italy), containing 8 μL of sulfur hexafluoride/mL was injected intravenously. The size distribution of SonoVue® is described in Schneider, M., 1999. Characteristics of SonoVue™. Echocardiography, 16, pp.743-746 26. In this article, the microbubbles are described as: " The bubble concentration of SonoVue^TM^ is between 100 and 500 million per ml. The mean bubble diameter is 2.5 pm and more than 90% of the bubbles are smaller than 8 pm". The mechanical index was reduced to 0.08 to preserve microbubble integrity during acquisition. The frame rate and duration depended on the kidney depth and the length of the patient's breath hold, respectively. It should be noted that while SonoVue® is routinely used in renal imaging within clinical settings, its application remains off-label.

### sULM post-processing

CEUS loops were divided into blocks of 200 frames each. A succession of steps was then applied on each block to generate a ULM density map (number of microbubbles tracks accumulated per pixel). The first step involved bandpass temporal filtering (frequency ranging from 0.5-5.5 Hz) in separating the datasets into high-velocity filtered microbubbles and slower non-filtered microbubbles. Indeed, sULM uses a dual filtering, dual localization, and dual tracking system to track both fast and slow microbubbles. Thus, slow microbubbles are localized and tracked thanks to “slow parameters” detailed above. There is no filter applied to CEUS acquisition; in fact, classical clinical CEUS acquisitions are based on line-by-line contrast pulse sequence (CPS) which is based on non-linear microbubble behavior; therefore, it enhances all the microbubbles regardless of their speed 27, and the localization/tracking is adapted to track small displacement. On the other hand, fast microbubbles are filtered with a bandpass filter to enhance fast-moving microbubbles (applied on the CEUS data with the same cutoff for all patients), and localization/tacking parameters are adapted to follow big displacement.

Then, we obtained microbubble super-resolved positions in both lateral and axial dimensions using targeted regional maxima on the filtered image, i.e., 2D Gaussian filtering 28. Microbubbles were then tracked using the Hungarian algorithm and simple tracker toolbox in Matlab 29. These steps were repeated for each block to obtain a ULM density map. We use two different sets of microbubble tracking parameters (detailed above) to establish 2 density maps, which we combine into a composite map (with slow flows in violet and fast flows in green). Moreover, the sULM technique enables the classification of microbubbles in the whole organ vasculature, including glomeruli as previously demonstrated 20.

The sULM parameters used in the study, differences in the processing pipeline of sULM and ULM** (Figure S3),** the advantage of tracking glomeruli with sULM rather than temporal accumulation of clip images (i.e. Power Doppler) (**Figure S4**), and double post-processing “classification” are detailed in the Supplemental Materials Section.

### Glomeruli detection and count

We were able to carry out a count of glomeruli on the ULM map using the normalized distance metric 20, which represents cumulative distance covered by each track divided by the distance between the first and last points of the track 30,31. Glomeruli were then targeted by selecting the points greater than the 90th percentile of the filtered normalized distance grid from a 2D Gaussian filtering 20. Tumors, pseudotumors, and the normal renal cortex adjacent to the lesion (control) were manually segmented. The number of glomeruli were normalized by this segmented area (cm^2^).

sULM algorithms used for vascular reconstruction are available in the following GitHub repository: https://github.com/EngineerJB/akebia 20. Akebia, a standalone application useable without MATLAB license, is available in the same repository.

### sULM metrics: normalized speed and dispersity

The normalized speed is defined as the distance divided by time. The dispersity corresponded to the number of times a track goes in the same direction, taking the rounded location of each track, with a tolerance of plus or minus 20°, divided by the number of points constituting the track 20. We compared these 2 metrics between tumors, pseudotumors, and normal renal cortex.

**Figure S5** shows an explanation of these metrics.

**Figure S6** shows conventional Doppler, CEUS, sULM density, and sULM velocity maps, and **Figure S7** illustrates the different stages of sULM process according to Denis *et al.* 20.

### Statistical analyses

Statistical analyses were performed using JAMOVI (version 2.3.26). We utilized JAMOVI's capabilities to calculate descriptive statistics, including the means, standard deviations (SDs), and ranges. For inferential statistics, we conducted an ANOVA test to determine if there were any statistically significant differences between the means of the 3 independent groups: normal renal cortex, pseudotumors, and tumors. When an ANOVA test identified significant differences, a Tukey post-hoc test was employed to pinpoint specific group differences. These analyses adhered to a 95% confidence interval and assumed a Gaussian distribution, which was confirmed by the Shapiro-Wilk test. The levels of significance in our results were defined as follows: 'ns' indicating a nonsignificant difference where P > 0.05, and significant levels were denoted by P ≤ 0.05.

## Results

### Patients, lesions, and CEUS characteristics

The average age of patients with tumors was 54 years [range: 30-68], while patients with pseudotumors had an average age of 61 years [range: 47-68]. The gender distribution was 1 woman for every 2 men in the tumor group, and 2 women for every 3 men in the pseudotumor group. One patient exhibited 2 pseudotumors. The mean eGFR was 32 [range: 21-98] for tumor patients and 70 [range: 21-98] for pseudotumor patients. One patient in the tumor group required dialysis.

**Table 1** summarizes patient characteristics.

Four of the 6 tumors were determined through pathological analysis to be malignant, including 1 clear cell renal cell carcinoma, 1 papillary renal neoplasm with reverse polarity, 1 renal tubule mucinous spindle cell carcinoma, and 1 papillary renal cell carcinoma. The remaining 2 were renal angiomyolipomas with typical CT features. The average diameter of the tumors was 30 mm [range: 17-51]. Among the 6 pseudotumors, 5 were confirmed as hypertrophy of the column of Bertin using both enhanced CT and MRI. Key indicators include tissue continuity with the renal cortex and similar density or signal intensity as the surrounding renal tissue. These columns exhibit contrast enhancement patterns akin to normal renal cortex on CT and MRI post-administration of a contrast agent. They are also distinguished from pathological lesions by their lack of malignancy signs 32. One was biopsied and identified as focal compensatory hypertrophy. The average diameter of these pseudotumors was 30 mm [range: 16-41].

**Table 2** summarizes lesion characteristics.

Regarding CEUS, the mean frame rate for tumor was 39 Hz [range: 32-43], whereas it was 44 Hz [range: 32-56] for pseudotumor. The average depth of the lesions was 23 mm [range: 13-39] for tumors and 42 mm [range: 32-68] for pseudotumors. The mean duration of the CEUS loop was 21 seconds [range: 17-26] for tumors and 19 seconds [range: 17-40] for pseudotumors.

**Table 3** summarizes CEUS characteristics.

### Glomeruli account

**Figure 1:**
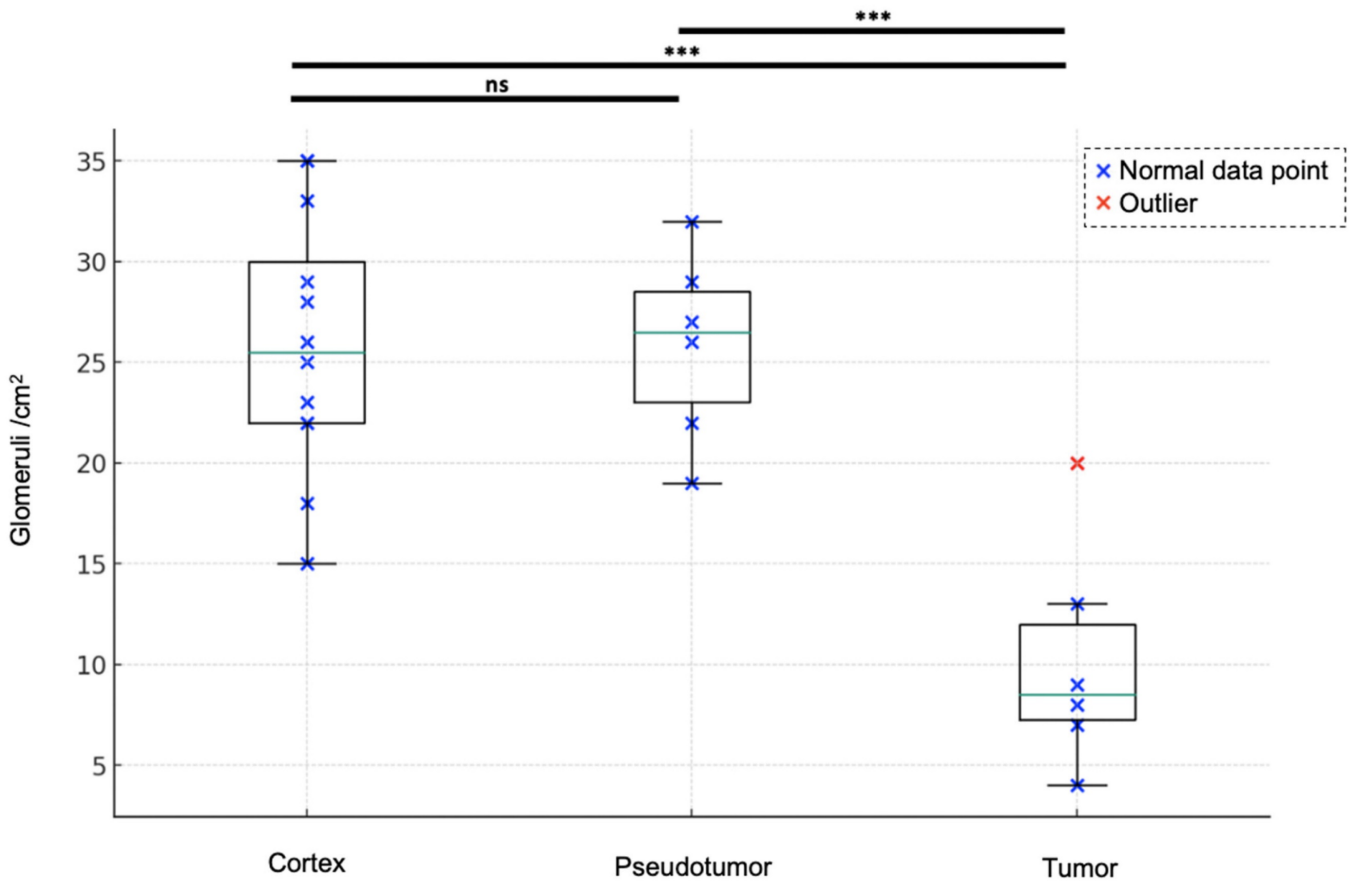
** Glomeruli account for the kidney cortex, pseudotumors, and tumors.** Renal tumors exhibited fewer detected glomeruli paths (mean of 10 ± 6 /cm2 [range: 4-20]) compared to both renal pseudotumors (mean of 26 ± 5 /cm2 19-32) and kidney cortex (mean of 26 ± 6 /cm2 15-35). ANOVA analysis: F-value of 16.21 and p-value < 0.0001. Tukey test: no significant difference between the cortex and pseudotumor groups (p = 0.6018). Statistical difference between cortex and tumor groups and between pseudotumor and tumor groups was p < 0.001 for both. ***: p < *0.001; ns = not statistically significant*.

**Figure 2:**
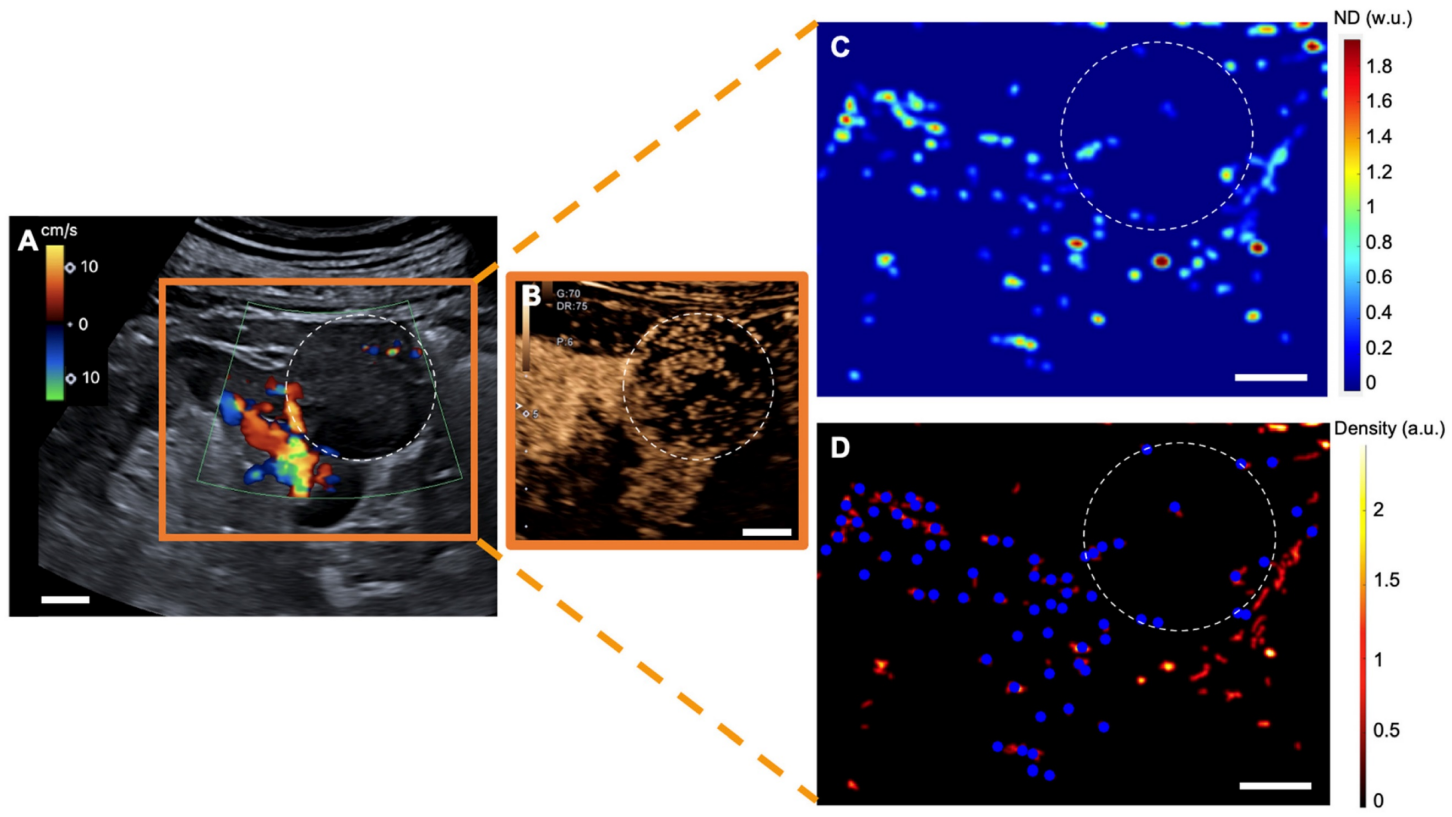
** sULM of clear cell renal cell carcinoma (ccRCC) (patient 1).** A & B. US Doppler image (A) and CEUS (B) showing an exophytic renal tumor (ccRCC) (*white-dotted area*). C & D show normalized distance metrics. (C) This metric enhances glomerular behavior, highlighting the detected glomeruli in blue points on the density map; (D) Note the presence of glomerular paths in the renal cortex and some artifacts mimicking glomeruli in the renal tumor. (The traces projected on the grid are displayed in red on the image. The colorbar corresponds to the count of the number of bubbles per pixel. Scale bars indicate 10 mm. *ND= normalized distance; w.u.= without units; a.u. = arbitrary units*.

**Figure 3:**
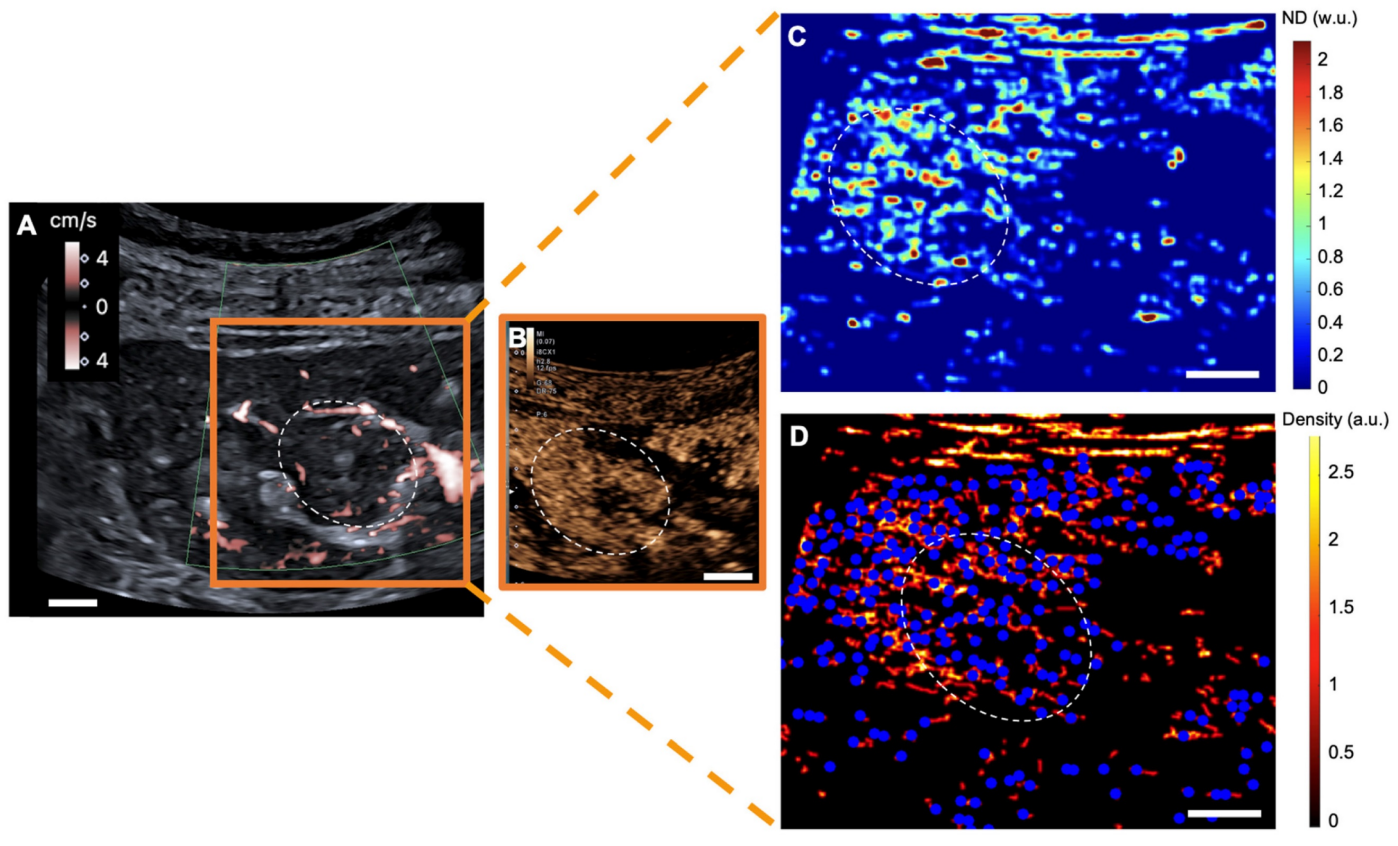
** sULM of hypertrophy of the column of Bertin (patient 7).** A & B. Superb microvascular imaging Doppler image (A) and CEUS (B) showing an endophytic renal pseudotumor (hypertrophy of column of Bertin) (*white-dotted area*). C & D show normalized distance metrics. (C) This metric enhances glomerular behavior, highlighting the detected glomeruli in blue points on the density map. (D) Note the presence of glomerular paths in the renal cortex and the pseudotumor. (The traces projected on the grid are displayed in red on the image. The colorbar corresponds to the count of the number of bubbles per pixel. Scale bars indicate 10 mm. *ND= normalized distance; w.u.= without units; a.u. = arbitrary units*.

With a mean of 10 ± 6 /cm^2^ [range: 4-20], renal tumors exhibited fewer detected glomeruli paths than did both renal pseudotumors (mean of 26 ± 5 /cm^2^ [19-32]) and kidney cortex (mean of 26 ± 6 /cm^2^ [15-35]). While statistical analysis indicated no significant difference in detected glomeruli paths between kidney cortex and renal pseudotumors (p = 0.60), there was a statistical difference in detected glomeruli paths between kidney cortex and tumors (p < 0.001), as well as between pseudotumors and tumors (p < 0.001) (**Figure 1**). Detailed sULM metrics are summarized in **Table 4**. Representative examples of renal tumors and pseudotumors are provided in **Figures 2** and **3**, respectively.

### Other sULM metrics

**Figure 4:**
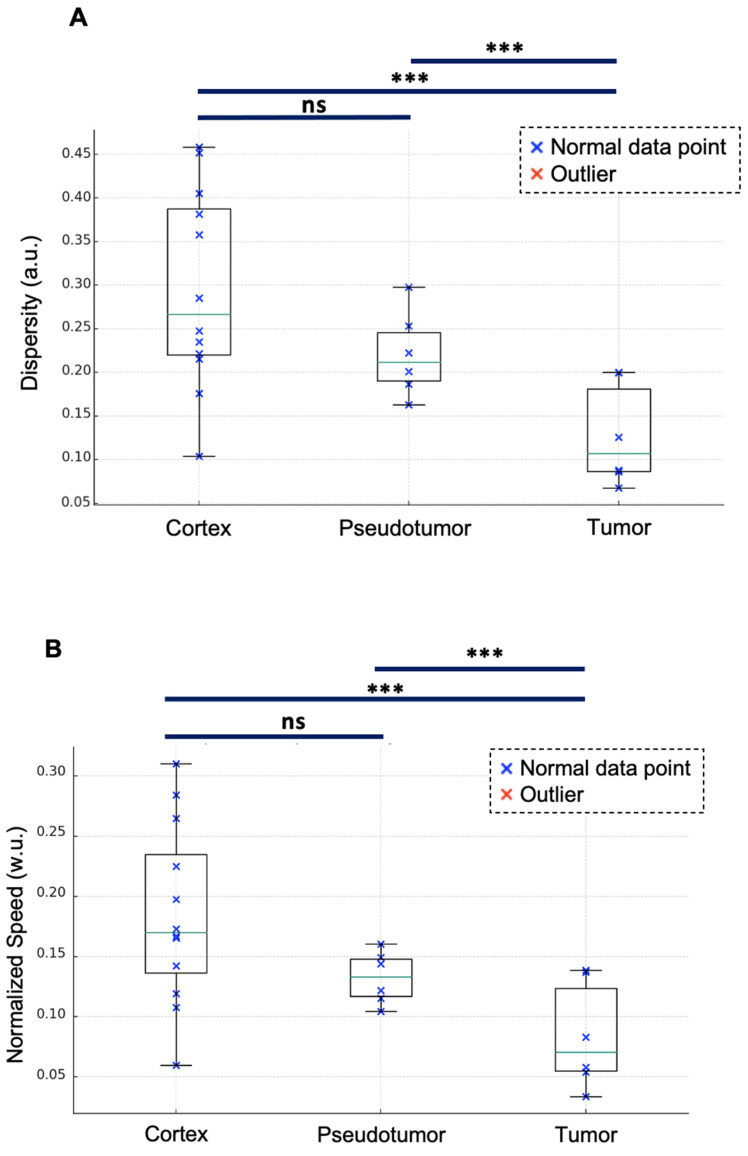
** Dispersity and normalized speed for the kidney cortex, pseudotumor, and tumor.** Renal tumors exhibit lower dispersity (mean of 0.13 ± 0.06 a.u. [range: 0.07-0.20]) compared to renal pseudotumor (mean of 0.22 ± 0.05 a.u. [0.16-0.25]) and kidney cortex (mean of 0.30 ± 0.11 a.u. [0.10-0.38]). ANOVA analysis: F-value of 6.89 and p-value of 0.005. Tukey test: no significant difference between the kidney cortex and pseudotumor groups (p = 0.513). Statistical difference between kidney cortex and tumor groups (p = 0.0012), and between pseudotumor and tumor groups (p = 0.0389). Renal tumors exhibited lower normalized speed (mean of 0.08 ± 0.04 w.u. [range: 0.03-0.17]) compared to renal pseudotumor (mean of 0.14 ± 0.02 w.u. [0.12-0.16]) and kidney cortex (mean of 0.18 ± 0.07 w.u. [0.11-0.28]). ANOVA analysis: F-value of 6.32 and p-value of 0.0065. Tukey test: no significant difference between the kidney cortex and pseudotumor groups (p = 0.272). The statistical difference between kidney cortex and tumor groups was p = 0.0014 and the statistical difference between pseudotumor and tumor groups was p = 0.0497. ***: p < 0.001; *ns=not statistically significant. w.u.= Without Units; a.u. = arbitrary units*.

With a mean of 0.13 ± 0.06 a.u. [range: 0.07-0.20], renal tumors exhibited lower dispersity than did both renal pseudotumors (mean of 0.22 ± 0.05 a.u. [0.16-0.25]) and kidney cortex (mean of 0.3 ± 0.1 a.u. [0.1-0.4]). While statistical analysis indicated no significant difference in dispersity between kidney cortex and renal pseudotumors (p = 0.513), there was a statistical difference between the dispersity of kidney cortex and tumors (p = 0.0012), as well as between pseudotumors and tumors (p = 0.0389) (**Figure 4A**).

Renal tumors exhibited lower normalized speed (mean of 0.08 ± 0.04 w.u. [range: 0.03-0.17]) than did both renal pseudotumors (mean of 0.14 ± 0.02 w.u. [0.12-0.16]) and kidney cortex (mean of 0.18 ± 0.07 w.u. [0.11-0.28]). Statistical analysis indicated no significant difference in normalized speed between kidney cortex and renal pseudotumors (p = 0.272). However, there was a statistical difference between both kidney cortex and tumors (p = 0.0014), as well as between pseudotumors and tumors (p = 0.0497) (**Figure 4B**). We also quantified the absolute speeds (without normalization). With a mean of 2 ± 1 cm/s [range: 1-4], renal tumors exhibited lower speed than did renal pseudotumors (mean of 3.4 ± 0.8 cm/s [2.3-4.6]) and kidney cortex (mean of 4 ± 1 cm/s [range: 2-6]). Statistical analysis indicated no significant difference in speed between the kidney cortex and renal pseudotumors (p = 0.1871), however there was a statistical difference between the speed of kidney cortex and tumors (p = 0.0011), as well as between the speed of pseudotumors and tumors (p = 0.0237).

Detailed dispersity and normalized speed values are summarized in **Table S2**.

## Discussion

Renal tumors exhibit fewer glomeruli paths per square centimeter (mean: 10 ± 6 /cm² [range: 4-20]) than do both pseudotumors (mean: 26 ± 5 /cm² 19-32) (p < 0.001) and kidney cortex (mean: 26 ± 6 /cm² 15-35) (p < 0.001). These results underline the ability of sULM to distinguish renal pseudotumors, the site of glomeruli, from tumors that do not have glomeruli 33. Furthermore, tumors displayed lower normalized speeds and dispersity values than did both renal cortex (p = 0.0012 and p = 0.0014) and pseudotumors (p = 0.0389 and p = 0.0497).

In addition to having a lower density of glomeruli in tumors, the dispersity and normalized speed results observed in the renal cortex align with our previous study, in which we showed that the glomeruli tracks were both significantly slower and less dispersed than those of the main vessels of the renal cortex 20. The speed results without normalization are consistent with those in the literature 34,35. Attributable to the presence of glomeruli, these metrics are similar between pseudotumors and renal cortex (healthy tissue), but are notably diminished in tumors. Several pathophysiological mechanisms may contribute to this diminution. Vascular anomalies, characterized by aberrant and tortuous vessel architecture, disrupt conventional hemodynamics 36. Further, heterogeneous perfusion patterns, wherein disparate regions of the tumor exhibit varying blood supply, contribute to localized reductions in blood flow 37. Increased interstitial pressure, frequently noted in tumors, may also compress vasculature, thereby impeding circulation 38. Hypercoagulability, a common complication in cancer patients, heightens the risk of thrombosis in microvessels 39. Rapid cellular proliferation within tumors can induce hypoxia and acidosis, which are detrimental to both vascular function and circulatory efficiency 40. The immune-mediated inflammatory response to the tumor further exacerbates vascular congestion and dysfunction 41. Additionally, the physical presence of tumor cells can directly obstruct microvascular channels. These multifactorial influences render the vascular milieu of tumors complex and often compromised 42. Finally, tumor-induced neoangiogenesis may lead to entrapment or stagnation of microbubbles in CEUS, thereby obscuring anticipated differences in remanence.

To date, most super-resolution ultrasound applications are concentrated on preclinical animal models and pilot clinical studies 43. Our pilot study introduces a clinically significant application of sULM's ability to distinguish renal pseudotumors from tumors. This approach may be especially beneficial when traditional imaging techniques, such as enhanced CT or MRI, are impractical or have potential risks. This is particularly common for patients with severe chronic renal failure or biopsy-related risks.

While this study's findings are promising, the study design has several limitations. First, this study represents an initial exploration into the potential of sULM for differentiating renal tumors from pseudotumors. The limited sample size is a significant constraint, which affects the generalizability of the results. With a small patient cohort, this work should be considered a preliminary proof of concept rather than definitive evidence. Further investigations with larger, more diverse patient populations are necessary to validate these early results and to better understand the diagnostic performance of sULM in clinical settings. Moreover, the co-localization of the glomeruli and the specific kinetic of the microbubbles observed in sULM was demonstrated only indirectly in animals. Direct demonstration would require co-registering deep microscopy techniques, such as two-photon microscopy and sULM, which remains challenging. Additionally, the patient cohort was small, with a focus on hypertrophy of the column of Bertin and focal compensatory hypertrophy. Further research with larger and more homogenous patient groups and a broader spectrum of pseudotumors, such as Junctional Parenchymal Defect and focal infection disease, is crucial to confirming these trends and establishing the reproducibility of sULM in various pseudotumors and patient populations. Additionally, this study's figures for the glomerular count within the cortex were lower than the real physiological number 44,45, though consistent with other sULM results in the literature. This may be linked to loops of limited duration and exploration in 2 dimensions, which does not allow all glomeruli to be imaged 20,21. Further, the presence of glomerulus paths in tumors could be artefactual, particularly linked to their heterogeneous neovascularization, as well as out-of-plane paths due to the 2D imaging [20). Some glomerular paths in the renal cortex can be linked to artifacts, as is the case with pseudotumors. The advancement of 3D imaging techniques could benefit similar research plans in the future by improving the ability to capture an organ's complete volume and thereby offering both improved accuracy and reduced artifacts 13,15,46,47. Then, in our view, the glomeruli-linked microbubble behavior is specific and understandable based on our previous studies in animals where glomeruli were automatically segmented in rat's kidney with 3D sULM 48 and transplanted kidneys. Although the other metrics such as speed and dispersity display statistically significant difference between diseases and healthy tissue, we have not built a full understanding of the relation between these metrics. Thus, we chose to show them as secondary results and restrict the interpretation of these results. Moreover, the masks of tumors, pseudotumors, and renal cortex were segmented by hand in this study. Then is a well-established technique for characterizing various kidney lesions, with numerous studies demonstrating its clinical utility. Spiesecke *et al.* 49 showed that CEUS correctly identified 8 out of 9 neoplastic lesions, missing only one oncocytoma among the 32 included patients. Irregular vessel structure (88.9% vs. 13.0%, P = 0.007) and hyperenhancement (66.6% vs. 17.4%, P = 0.031) were significantly more common in neoplasic lesions compared to developmental pseudotumors. Compared to histopathology, CEUS demonstrated a sensitivity of 89% (95% CI 57-98), a specificity of 96% (95% CI 80-99), a positive predictive value of 89% (95% CI 57-98), and a negative predictive value of 96% (95% CI 79-99) for ruling out renal malignancy in developmental pseudotumors. These findings are consistent with McArthur *et al.* 50, who also highlighted the advantage of CEUS's lack of nephrotoxicity. Similarly, Mazziotti *et al.* 4 demonstrated that CEUS concordance with CT and MRI characterizing of all 24 pseudotumors deemed ambiguous at conventional and power Doppler US. CEUS provides an immediate alternative to referring patients for CT or MRI when B-mode US results are unclear. Renal pseudotumors with regular vascular architecture remain isoechoic to normal renal parenchyma in all enhancement phases following the administration of US contrast agents. In contrast, renal tumors typically exhibit distinct contrast enhancement patterns, such as early enhancement in the arterial phase or late wash-out phase, corresponding to about 90 to 95% of cases 49,51-53. Our study was designed as a pilot to explore the potential of sULM in cases where conventional US and CEUS were inconclusive. In our retrospective analysis, we applied sULM specifically to such challenging cases; therefore, we could not conduct a direct comparative study between US, CEUS, and sULM within this dataset. The rationale for using sULM in this context stems from its unique ability to visualize glomerular capillaries, offering insights that are not accessible with conventional methods. While CEUS provides a visualization of macrocirculation, sULM enables a detailed view of the functional unit network, which may offer additional diagnostic information in ambiguous cases where CEUS alone is insufficient. Moreover, sULM could be particularly useful in cases where the injection of CT-scan contrast agents is contraindicated, such as in patients with renal insufficiency. A direct comparison between CEUS and sULM would be valuable to demonstrate the added diagnostic value of sULM over standard CEUS. However, given the retrospective nature of our study and the specific inclusion criteria, such a comparison was not feasible in our current analysis. A prospective study comparing US, CEUS, and sULM in a well-defined cohort of patients, including those where CEUS results are inconclusive, would be an excellent next step to rigorously evaluate the added benefits of sULM and complete this first pilot study. Then, were able to successfully perform sULM on every patient included in this study. However, it is important to note that sULM requires high-quality CEUS acquisition. In a larger study with more patients, it is likely that not all scans would meet the necessary quality criteria, and sULM might not have been feasible for every patient. For example, in Bodard *et al.* 21, among 15 patients, sULM of native kidneys could not be performed in 3 cases (2 due to the inability to achieve breath-holding and 1 due to the kidney being positioned too deep (>8cm)). Moreover, the sULM process requires the absence of motion, which necessitates that patients hold their breath during the acquisition of the scanning section. Regarding the specific context of native kidneys, their anatomical position allows the operator to stabilize their hand on the patient's abdomen during acquisition, helping to minimize movement. However, it is important to note that slight residual motion may still occur, and breath-holding is not a feasible solution in routine clinical practice and can be challenging for some patients. The breath-hold duration can be reduced by increasing the ultrasound frame rate. Although the operator can attempt to optimize frame rate acquisition by adjusting settings, reducing the acoustic window, or decreasing the depth of exploration, these adjustments are still constrained by the capabilities of current clinical ultrasound scanners. The research community has actively proposed and developed various methods to address this challenge. These solutions range from filtering techniques and ultra-fast development, for which an acquisition of only a few seconds allows a complete mapping to be obtained 17 to more advanced approaches such as sparsity-based algorithms 54,55 and deep learning methods 56-61. Additionally, tissue motion correction techniques could help partially mitigate this limitation 62. However, CEUS sequence is specifically intended to maximize the detection of both slow- and fast-moving microbubbles, thus preserving the detailed vascular mapping required. Applying a filter for slow-moving microbubbles could lead to the unintended exclusion of quasi-static bubbles, potentially compromising the depiction of subtle microvascular structures such as glomeruli. Accelerating ULM remains an active area of research, and we anticipate that solutions for faster and more robust imaging will emerge in the near future. Finally, given that patients with renal failure of glomerular origin possess abnormal glomeruli, there is a critical need to evaluate how these abnormalities might influence the accuracy of sULM in detecting glomeruli. The current technique may count remaining glomeruli, including those that are abnormal, and this capability requires further detailed investigation.

The potential of sULM extends beyond renal pseudotumor differentiation. Future research avenues could encompass tumor characterization, monitoring of treatment response, assessment of angiogenesis, and guidance of treatment planning. Continued advancements in image analysis algorithms and computational techniques hold the potential to further optimize sULM's ability to detect subtle tissue variations and perform real-time analysis, thereby bolstering its clinical utility.

## Conclusion

In conclusion, our study presents a proof of concept highlighting the potential first clinical utility of sULM in differentiating renal tumors from pseudotumors based on glomerular presence. This could represent an advancement towards the integration of sULM into clinical practice. However, further prospective studies with larger patient cohorts are necessary to confirm these findings and establish sULM's diagnostic value more robustly.

## Supplementary Material

Supplementary information, figures and tables.

## Figures and Tables

**Table 1:** Patient characteristics

Patient number	Age (y)	Sex	BMI (kg/m²)	eGFR (mL/min/1.73m²)	Lesion number
	**54 [30-68]**	**2F/4M**	**26 [22-34]**	**77 [32-120]**	**Tumor**
1	30	F	24	84	1
2	67	M	26	72	2
3	59	M	26	66	3
4	64	M	22	32	4
5	68	M	25	90	5
6	35	F	34	120	6
	**61 [47-68]**	**2F/3M**	**28 [25-30]**	**70 [21-98]**	**Pseudotumor**
7	64	M	25	21	7
8	68	M	27	63	8
9	47	F	30	Dialysis	9
10	64	F	28	98	10
11	63	M	29	97	11 & 12

BMI: body mass index; eGFR: estimated glomerular filtration rate; F: female; M: male; y: year

**Table 2:** Lesion characteristics

Lesion type	Lesion number	Lesion diagnosis	Diagnostic method	Renal side	RENAL Score	Max. diameter (mm)
Tumor	1	Renal angiomyolipoma	CT/MRI	Right	10x	24
	2	Papillary renal cell carcinoma	Partial nephrectomy	Right	8a	42
	3	Clear cell renal cell carcinoma	Biopsy	Left	6p	21
	4	Papillary renal neoplasm with reverse polarity	Biopsy	Renal graft	7x	17
	5	Renal tubule mucinous spindle cell carcinoma	Biopsy	Right	5x	27
	6	Renal angiomyolipoma	CT/MRI	Left	8x	51
Pseudotumor	7	Hypertrophy of the column of Bertin	MRI	Renal graft	10x	35
	8	Hypertrophy of the column of Bertin	CT/MRI	Renal graft	9x	19
	9	Focal compensatory hypertrophy	Biopsy	Right	5p	41
	10	Hypertrophy of the column of Bertin	CT/MRI	Right	9a	19
	11	Hypertrophy of the column of Bertin	CT	Right	7p	25
	12	Hypertrophy of the column of Bertin	CT	Left	6a	16

CT: computed tomography; MRI: magnetic resonance imaging

**Table 3:** Contrast-enhanced ultrasound characteristics

Lesion number	Frame rate (fps)	CEUS loop duration (s)	Depth (mm)	Time after SonoVue injection (min : s)
**Tumor**	**39 [32-43]**	**21 [17-26]**	**23 [13-39]**	**03:43 [02:40-05:23]**
1	43	20	18	02:40
2	32	21	26	05:23
3	43	20	27	04:02
4	39	26	15	03:05
5	43	17	13	03:36
6	32	23	39	02:42
**Pseudotumor**	**44 [32-56]**	**19 [17-40]**	**42 [32-68]**	**02:25 [01:15-03:45]**
7	49	20	35	01:29
8	32	40	32	03:33
9	56	10	68	03:45
10	39	10	36	01:15
11	43	17	38	02:10
12	43	19	40	02:05

CEUS: contrast-enhanced ultrasound; Fps: frames per second

**Table 4:** Sensing ultrasound localization microscopy metrics

	Lesion			Kidney cortex	
CEUS loop duration (s)	Lesion number	Surface mask (cm^2^)	Number of glomeruli detected/cm^2^ mean ± SD [range]	Surface mask (cm^2^)	Number of glomeruli detected/cm^2^ mean ± SD [range]
**21 [17-26]**	**Tumor**	**2.8 [1.5-4.7]**	**10 ± 6 [4-20]**	**1.3 [0.8-3.3]**	**26 ± 6 [15-35]**
20	1	2.9	7	0.9	18
21	2	4.7	9	1.2	23
20	3	1.5	20	0.9	25
26	4	1.7	13	3.3	22
17	5	2.2	8	1.0	22
23	6	3.8	4	0.8	15
**19 [17-40]**	**Pseudotumor**	**1.3 [1.1-1.7]**	**26 ± 5 [19-32]**		
20	7	1.1	29	0.8	35
40	8	1.2	27	1.3	29
10	9	1.7	22	0.9	26
10	10	1.2	26	0.6	35
17	11	1.3	19	0.7	28
19	12	1.5	32	0.6	33

SD: standard deviation

## Abbreviations

CT: computed tomography
MRI: magnetic resonance imaging
ULM: ultrasound localization microscopy
sULM: sensing ultrasound localization microscopy
CEUS: contrast-enhanced ultrasound
BMI: body mass index
eGFR: estimated glomerular filtration rate
SD: standard deviation
US: ultrasound
cm^2^: square centimeter
μm: micrometer
p: probability (used for statistical significance)
ccRCC: clear cell renal cell carcinoma
