# Supplementary material for: First clinical utility of sensing Ultrasound Localization Microscopy (sULM): identifying renal pseudotumors
Source: Theranostics. 2025 Jan 1;15(1):233–44. doi: 10.7150/thno.100897 (PMC11667240; doi:10.7150/thno.100897)
Supplement: Supplementary file 1 — Supplementary information, figures and tables. [file thnov15p0233s1.pdf]

SUPPLEMENTAL MATERIALS

sULM parameters

The sULM parameters used in the study are:

Slow-moving:

- *Intensity threshold for localization* = 140 (arbitrary units)
- *Max_linking_distance* (maximal distance between 2 bubbles in the same frame) = 3 pixels = 0.4 mm
- *Max_gap_closing* (number of frames jumped where 2 bubbles are not forcibly paired) = 2 frames = 0.05 sec
- *Min_length* (minimal duration of track) = 7 frames = 0.18 sec
- *Filt* (time filter cutoff frequency) = none

Fast-moving:

- *Intensity threshold for localization* = 100 (arbitrary units)
- *Max_linking_distance* = 10 pixels = 1.4 mm
- *Max_gap_closing* = 2 frames = 0.05 sec
- *Min_length* (minimal duration of track) = 5 frames = 0.13 sec
- *Filter* = [0.5 5.5]

Differences in the processing pipeline of sULM and ULM

The sULM processing pipeline differs from standard ULM by its enhanced ability to capture and analyze the slowest and non-linear microbubble movements, particularly those corresponding to the microcirculation within glomerular capillary beds. While standard ULM excels in providing microvascular mapping, where the vessels are generally quite linear, it is limited in distinguishing these very slow-moving microbubbles along such specific pathways. This is where sULM, a variation of ULM, makes its significant contribution. By utilizing microbubbles as sensors of their immediate environment, sULM increases the sensitivity of ULM. Through advanced filtering

methods and tracking algorithms, sULM can identify specific microbubble motion patterns associated with microscopic structures like glomeruli, enabling their direct visualization of what the ULM cannot do. **Figure S3** shows the differences between classical ULM post-processing and sULM post-processing treatments.

Advantage of sULM glomeruli tracking vs. temporal accumulation of clip images (i.e., Power Doppler)

Temporal accumulation has an inherent limitation by being limited within the diffraction of the ultrasound waves. In order to image a capillary bundle of order of 200 µm, super-resolution technique are crucial. The advantage of tracking glomeruli with sULM (Figure S4, on the right) rather than temporal accumulation of clip images (i.e. Maximum Intensity Projection, or with bandpass filter a Power Doppler) is that we can overcome the diffraction limit thanks to the microbubble localization step. Power Doppler only visualizes "large" structures (Figure S4, on the left), and could therefore not be used to visualize glomeruli. The advantage of tracking glomeruli with sULM rather than temporal accumulation of microbubble locations (SUSHI or SOFI methods) is that we have geometric (dispersity, distance metric) and temporal (velocity) information on microbubble tracking within the glomerulus. Indeed, by tracking the microbubble within the capillary bundle, we can establish velocity and dispersity metrics, something we wouldn't be able to do if we hadn't tracked the microbubble and just accumulated its intensity. As a demonstration, we compared an intensity-cumulated band-pass filtered cineloop with sULM below. The intensity coming from all microbubbles is confused in the first image, while the microbubbles are classified based on their specific kinetics in the second image.

Double post-processing "classification"

The sULM is based on a physiological a priori of flow velocity and microbubble behavior in the human kidney. We know that in the main arteries, the velocity of microbubbles in the blood is in the cm/sec to tens of cm/sec range [1]. while in the capillaries making up the glomeruli, the flow

is around a few mm/sec [2]. sULM, therefore, uses a dual filtering, dual localization, and dual tracking system to track both fast and slow microbubbles. So, we use two different sets of microbubble tracking parameters (detailed above) to establish 2 density maps, which we combine into a composite map (with slow flows in violet and fast flows in green). We call this double post-processing “classification”.

REFERENCES

1. Souza de Oliveira IR, Widman A, Molnar LJ, Fukushima JT, Praxedes JN, Cerri GG. Colour Doppler ultrasound: a new index improves the diagnosis of renal artery stenosis. Ultrasound Med Biol. 2000;26(1):41-7

2. Kang KY, Lee YJ, Park SC, Yang CW, Kim YS, Moon IS, et al. A comparative study of methods of estimating kidney length in kidney transplantation donors. Nephrology Dialysis Transplantation. 2007;22(8):2322-7

SUPPLEMENTARY FIGURES

Figure S1. Study flowchart

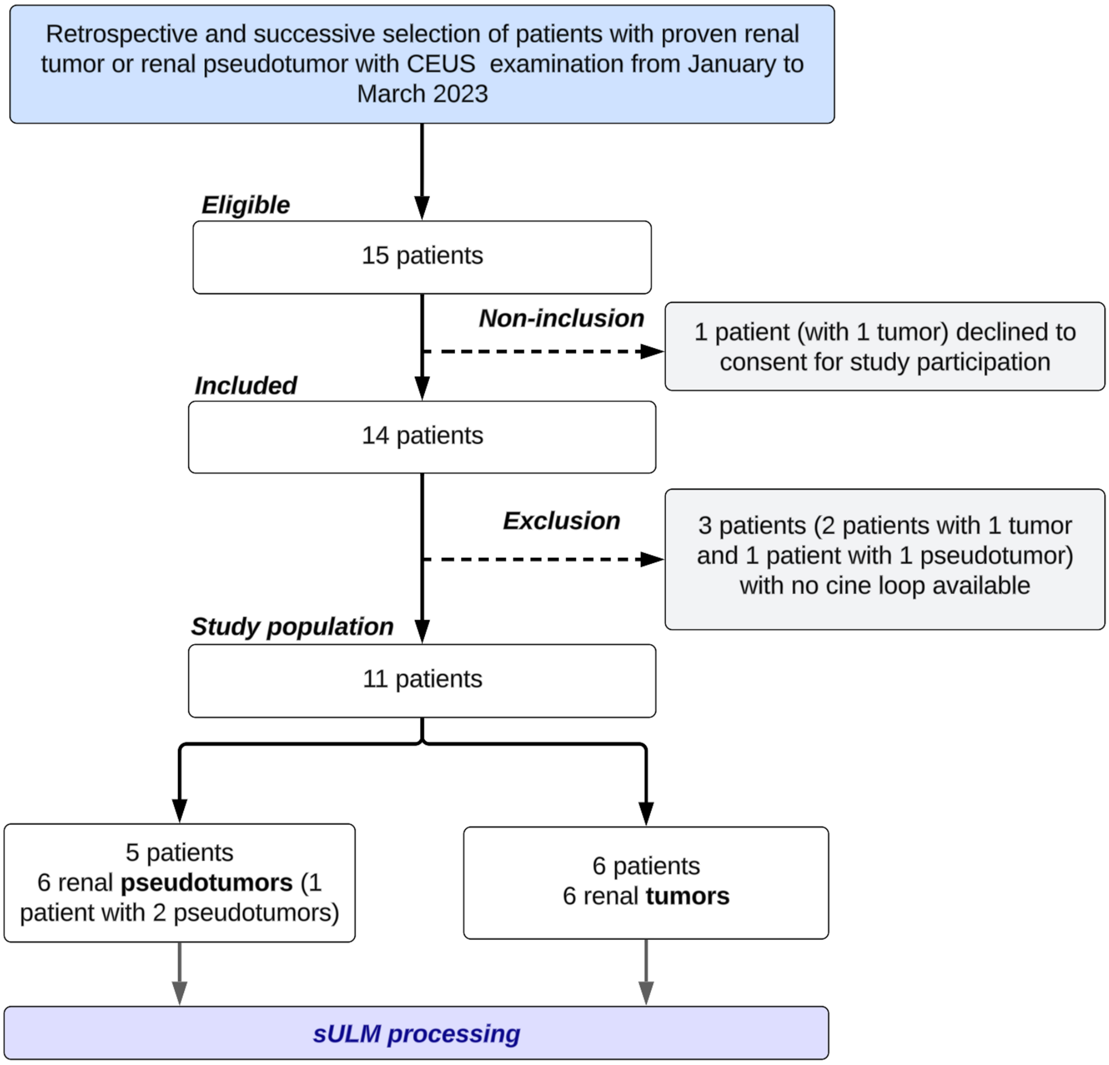

Figure S2. Example of diagnosis (gold standard) provided by imaging: Renal angiomyolipoma. The tumor demonstrates macroscopic fat (less than -20 HU, thin arrow) on non-enhanced CT (A), loss of signal (broad arrow) on fat saturation MRI (B) compared to the in-phase acquisition (C), and India ink artifact (arrowheads) at the interface between fat and non-fat components on out-of-phase (D) MRI

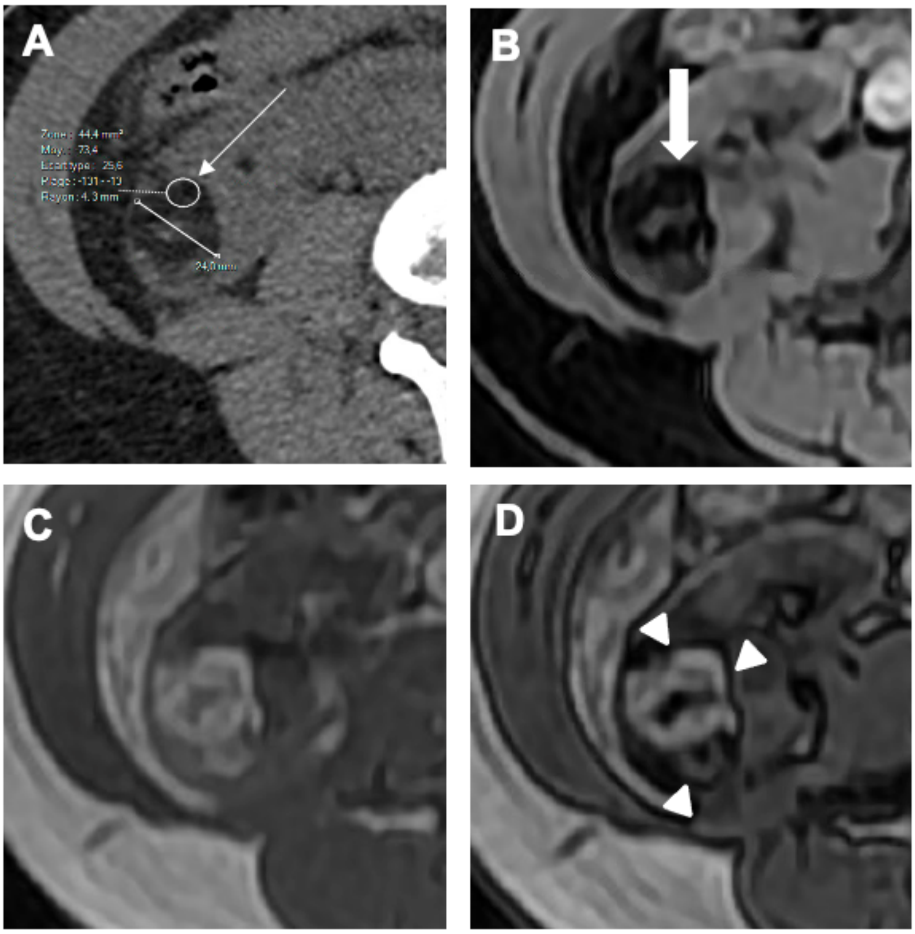

Figure S3. Differences between classical ULM post-processing treatment (C&D) and sULM post-processing treatment (A&B) in the same pseudo-tumor patient (patient n°7) from this study. Two different filters, localizations, and tracking are used to create sULM map (fast microbubbles in green and slow microbubbles in purple), whereas only one filter, localization, and tracking are used to create classical ULM map (only fast microbubbles in green). Classical ULM filters out glomeruli, whereas sULM can visualize slow flow displacement, including ones in glomeruli.

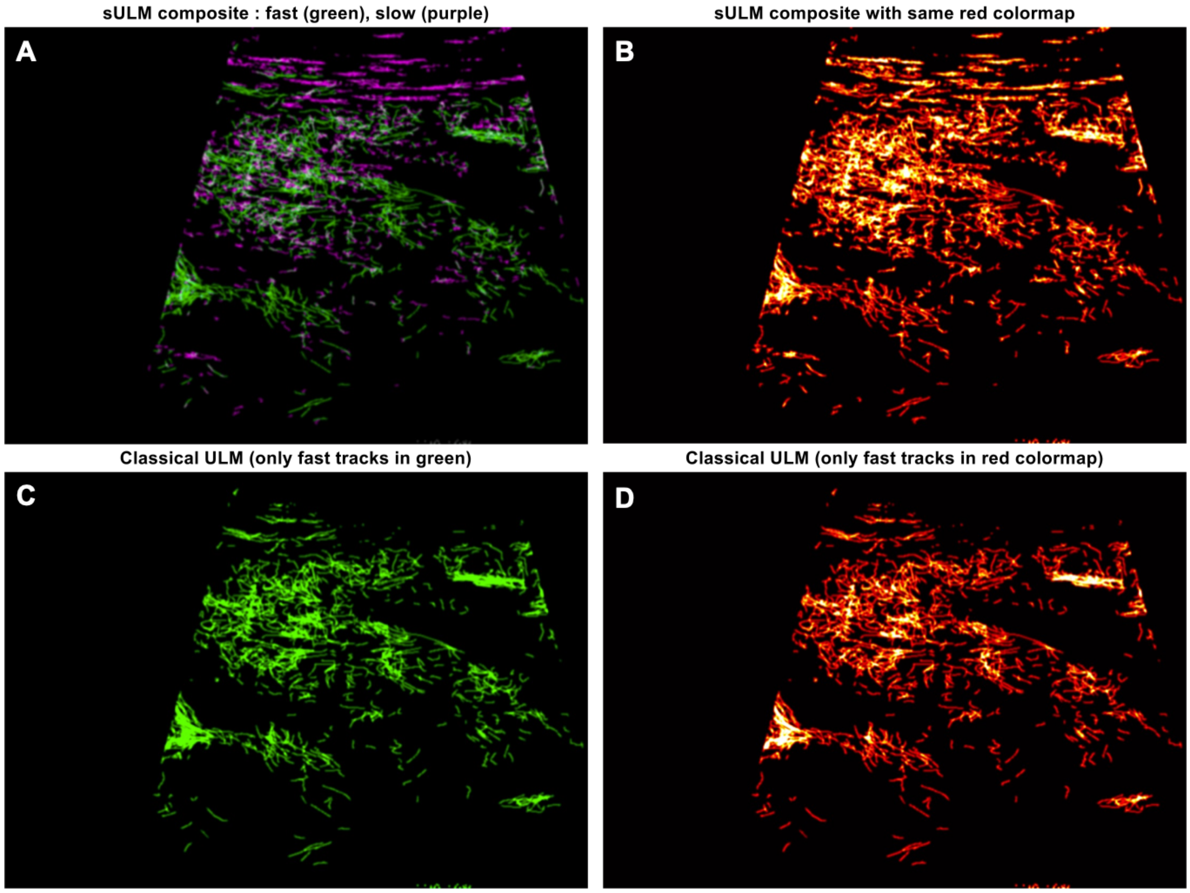

Figure S4. Differences between sULM (C&F) and temporal filtered clips accumulation, i.e. Maximum Intensity Projection (without filter) (A&D) and Power Doppler (with bandpass filter) (B&E), on a patient with a pseudo tumor included in this study. One can notice the clear absence of the glomeruli on the Maximum Intensity Projection (MIP) and on the Power Doppler compared to the sULM density map. Besides, tracking the microbubbles allow us to have a temporal (velocity metric) and geometrical information (dispersity) of the glomeruli blood flow.

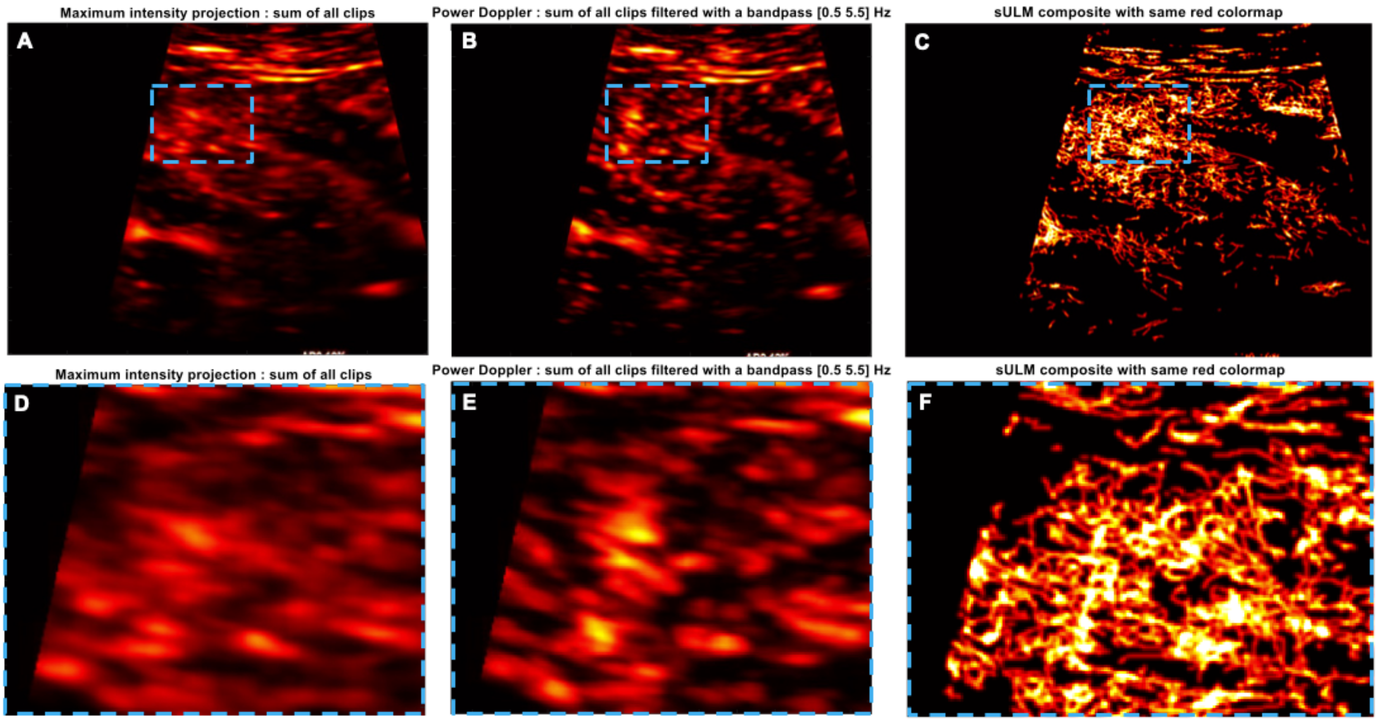

Figure S5. Metrics explanation: a temporal estimation of the tracks (velocity information) (A) and geometrical information of the tracks (dispersity metric) (B). Dispersity corresponds to the number of changes in the direction of the trace (>20°) divided by the number of points making up the trace. The speed corresponds to the distance covered by the microbubble divided by the time it took the microbubble to cover this distance.

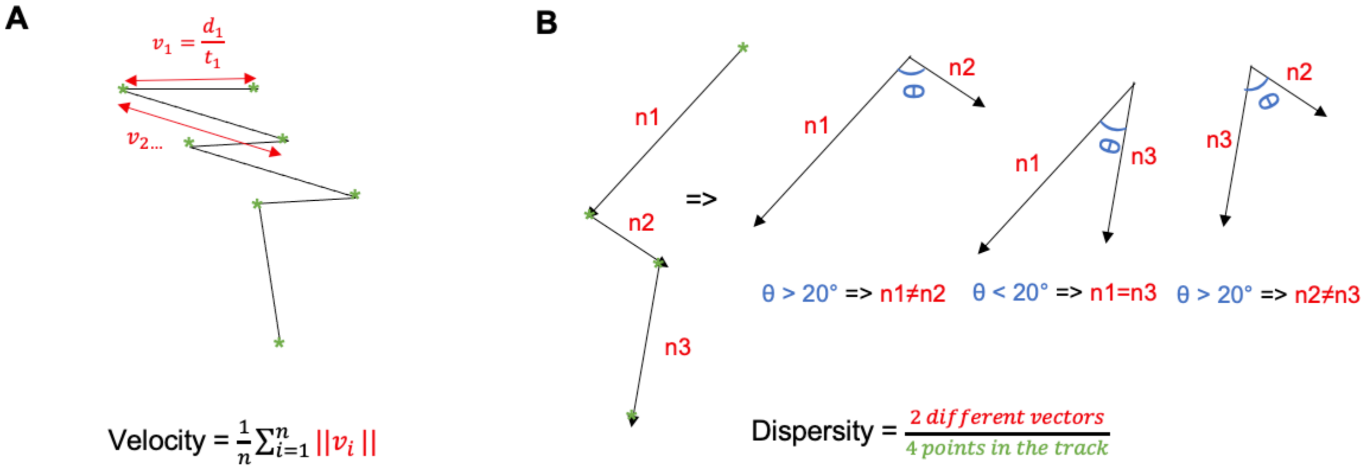

Figure S6. Conventional Doppler (A&E), CEUS (B&F), sULM density (C&G), and sULM velocity (G&H) maps of a renal tumor (A, B, C & D) and a renal pseudotumor (E, F, G & H) (Scale bars indicate 10 mm) [B, C & D: The acquisition time of CEUS has here been optimized to maximize the sensitivity to detect glomeruli in the mask (dots) which explains the absence of glomeruli detected in the adjacent kidney]

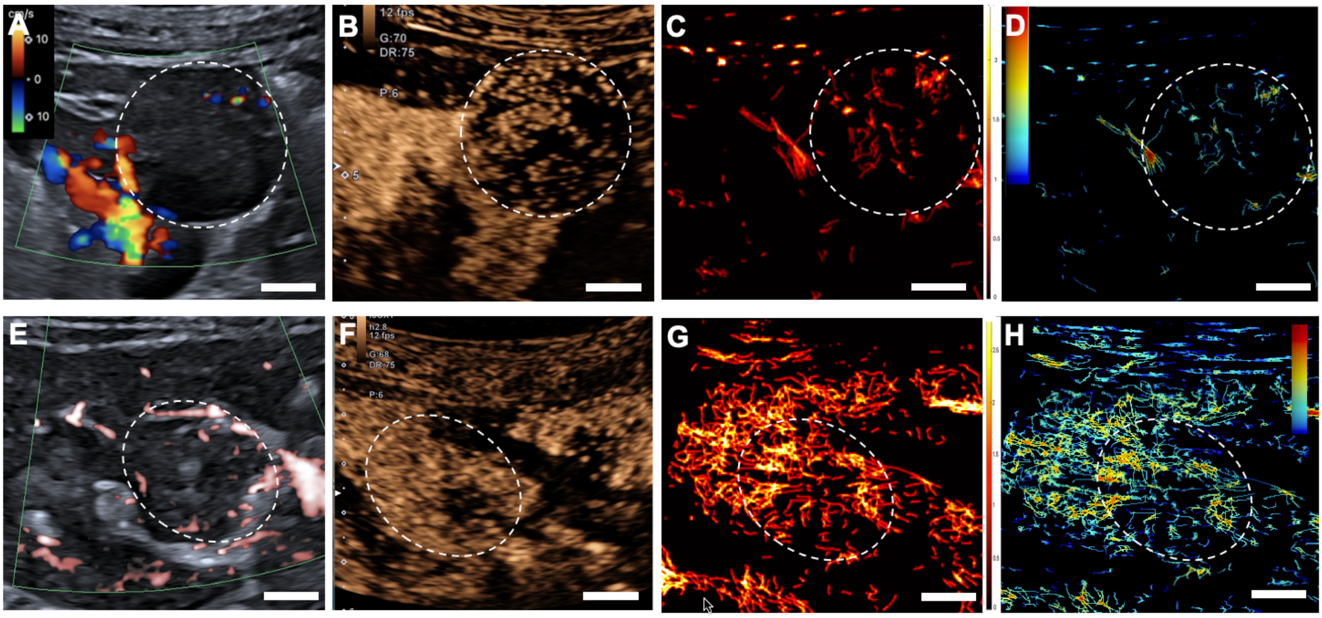

Figure S7. Different stages of sULM process (Scale bars indicate 10 mm)

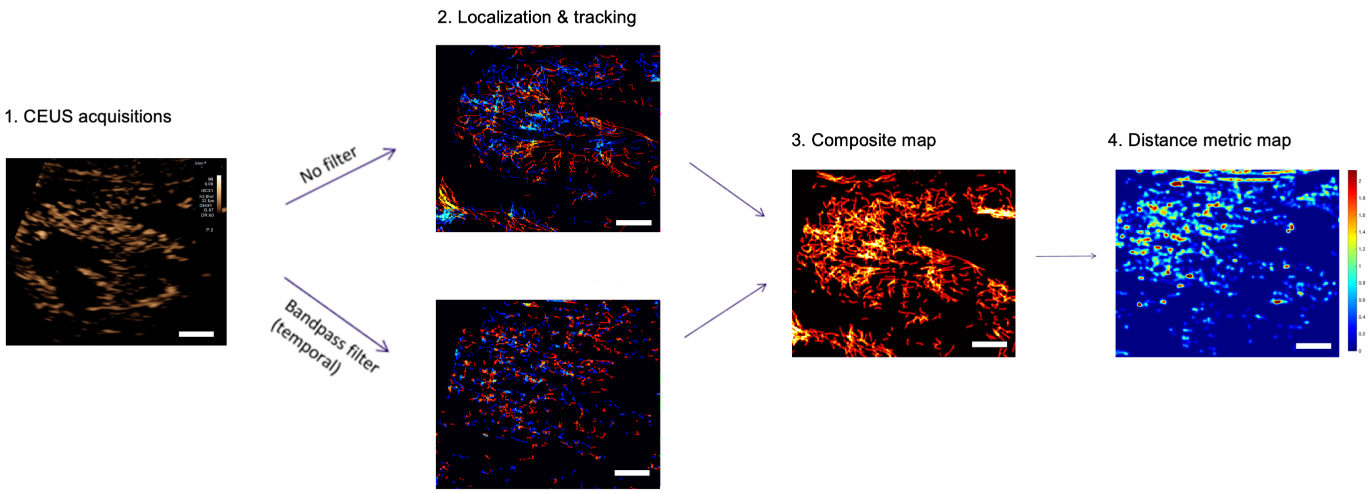

SUPPLEMENTARY TABLES

Table S1. Lesion characteristics in two-dimensional ultrasound and CEUS manifestations

Table S2. Dispersity and normalized speed

Table S1. Lesion characteristics in two-dimensional ultrasound and CEUS manifestations

Lesion number	2D-US with Doppler	CEUS
1	Mildly hyperechoic relative to the surrounding parenchyma. Doppler US with limited assessment	Ambiguous enhancement pattern. No definitive evidence of washout detected
2	Iso-echoic relative to the surrounding parenchyma. Indeterminate flow patterns on Doppler	Absence of washout. Indeterminate findings
3	Mildly hyperechoic relative to the surrounding parenchyma. Doppler US with limited assessment	Gradual enhancement pattern. No definitive evidence of washout detected
4	Mildly hyperechoic relative to the surrounding parenchyma. Doppler US with limited assessment	Ambiguous enhancement pattern. Indeterminate findings
5	Mildly heterogeneous echotexture. Vascularity not well visualized	No definitive evidence of washout detected. Inconclusive exploration
6	Mildly hyper-echoic compared to the surrounding parenchyma. No definitive flow patterns detected	No definitive evidence of washout detected. Inconclusive exploration
7	Mildly heterogeneous in echotexture. Equivocal Doppler US exploration	Progressive enhancement pattern. No definitive evidence of washout detected
8	Mildly hypoechoic relative to the surrounding parenchyma. Vascularity of the lesion not well visualized, no definitive flow patterns detected	Limited CEUS assessment. Enhancement pattern not well defined
9	Mildly heterogeneous in echotexture. Limited Doppler US assessment	Limited CEUS assessment. Enhancement pattern not well defined
10	Mildly hypoechoic. Vascularity not well visualized	Ambiguous enhancement pattern. Indeterminate findings
11	Mildly hypoechoic relative to the surrounding parenchyma. Doppler US findings are non-contributory	Limited CEUS assessment. Inconclusive exploration
12	Isoechoic or mildly hypoechoic relative to the surrounding parenchyma. No specific vascular patterns identified	Ambiguous enhancement pattern. No washout detected.

2D-US: two-dimensional ultrasound; CEUS: contrast enhanced ultrasound.

Table S2. Dispersity and normalized speed

Lesion number	Lesion		Cortex kidney	
	**Dispersity (a.u.)** mean±SD [range]	**Normalized speed (w.u.)** mean±SD [range]	**Dispersity (a.u.)** mean±SD [range]	**Normalized speed (w.u.)** mean±SD [range]
**Tumor**	**0.13 ± 0.06 [0.07-0.20]**	**0.08 ± 0.04 [0.03-0.17]**	**0.30 ± 0.11 [0.10-0.38]**	**0.18 ± 0.07 [0.11-0.28]**
1	0.09	0.06	0.38	0.17
2	0.07	0.03	0.25	0.17
3	0.20	0.14	0.29	0.20
4	0.20	0.17	0.10	0.06
5	0.13	0.08	0.18	0.11
6	0.09	0.05	0.41	0.28
**Pseudotumor**	**0.22 ± 0.05 [0.16-0.25]**	**0.14 ± 0.02 [0.12-0.16]**		
7	0.30	0.15	0.36	0.12
8	0.25	0.14	0.22	0.23
9	0.16	0.12	0.24	0.14
10	0.19	0.10	0.22	0.31
11	0.20	0.12	0.46	0.26
12	0.22	0.16	0.45	0.17

SD: standard deviation; a.u.: arbitrary units; w.u.: without units
